# (*E*)-3-[4-(Diphenyl­amino)­phen­yl]-1-(pyridin-2-yl)prop-2-en-1-one

**DOI:** 10.1107/S1600536811055036

**Published:** 2012-01-14

**Authors:** Cong-Bin Fan, Xiao-Mei Wang

**Affiliations:** a College of Chemistry, Chemical Engineering and Materials Science, Soochow University, Suzhou 215123, People’s Republic of China; b Jiangsu Key Laboratory for Environment Functional Materials, Suzhou University of Science and Technology, Suzhou 215009, People’s Republic of China

## Abstract

The title compound, C_26_H_20_N_2_O, belongs to a new family of organic two-photon absorption materials with triphenyl­amine and pyridine units. In the mol­ecule, the three benzene rings are arranged in a propeller-like fashion; the dihedral angles between the rings are 80.01 (14), 75.68 (13) and 56.93 (14)°. The pyridine ring is oriented at dihedral angles of 56.24 (14), 48.92 (15) and 22.02 (13)° with respect to the three benzene rings. Weak inter­molecular C—H⋯O hydrogen bonding is present in the crystal structure.

## Related literature

For applications of two-photon absorption compounds, see: Fan *et al.* (2012 ▶); He *et al.* (2008 ▶).
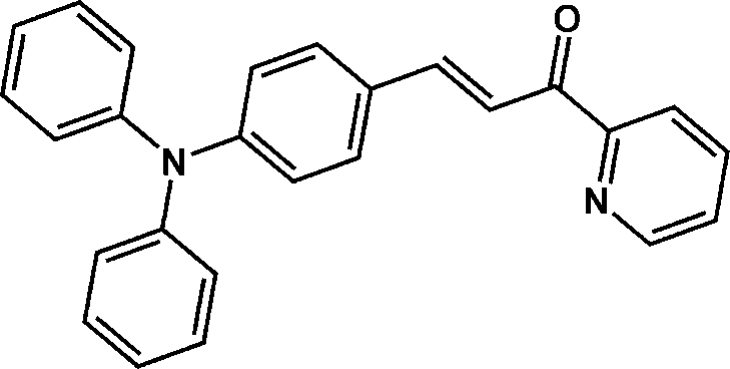


## Experimental

### 

#### Crystal data

C_26_H_20_N_2_O
*M* *_r_* = 376.44
Monoclinic, 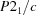

*a* = 12.0342 (3) Å
*b* = 17.4069 (4) Å
*c* = 9.4392 (2) Å
β = 91.757 (2)°
*V* = 1976.38 (8) Å^3^
*Z* = 4
Mo *K*α radiation
μ = 0.08 mm^−1^
*T* = 296 K
0.20 × 0.20 × 0.18 mm

#### Data collection

Bruker SMART 1000 CCD area-detector diffractometer
15074 measured reflections
3488 independent reflections
2210 reflections with *I* > 2σ(*I*)
*R*_int_ = 0.046

#### Refinement

*R*[*F* ^2^ > 2σ(*F* ^2^)] = 0.055
*wR*(*F* ^2^) = 0.167
*S* = 1.03
3488 reflections
263 parameters
H-atom parameters constrained
Δρ_max_ = 0.17 e Å^−3^
Δρ_min_ = −0.16 e Å^−3^

### 

Data collection: *SMART* (Bruker, 2007 ▶); cell refinement: *SAINT* (Bruker, 2007 ▶); data reduction: *SAINT*; program(s) used to solve structure: *SHELXTL* (Sheldrick, 2008 ▶); program(s) used to refine structure: *SHELXTL*; molecular graphics: *SHELXTL*; software used to prepare material for publication: *SHELXTL*.

## Supplementary Material

Crystal structure: contains datablock(s) global, I. DOI: 10.1107/S1600536811055036/xu5410sup1.cif

Structure factors: contains datablock(s) I. DOI: 10.1107/S1600536811055036/xu5410Isup2.hkl

Supplementary material file. DOI: 10.1107/S1600536811055036/xu5410Isup3.cml

Additional supplementary materials:  crystallographic information; 3D view; checkCIF report

## Figures and Tables

**Table 1:** Hydrogen-bond geometry (Å, °)

*D*—H⋯*A*	*D*—H	H⋯*A*	*D*⋯*A*	*D*—H⋯*A*
C5—H5⋯O1^i^	0.93	2.55	3.404 (3)	153
C6—H6⋯O1^ii^	0.93	2.58	3.505 (3)	179

Symmetry codes: (i) 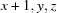; (ii) 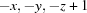.

**Fig. 1.**
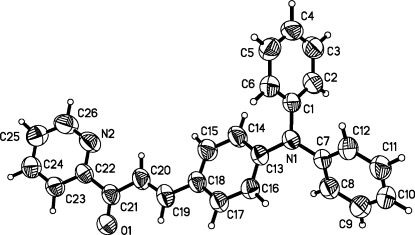
Molecular structure of the title compound with displacement ellipsoids drawn at the 30% probability level. H atoms are represented as small spheres of arbitrary radii.

## supplementary crystallographic information

### Comment

Organic materials with two-photon absorption (TPA) properties have attracted increasing attention due to their versatile potential applications in two-photon excited fluorescence (Fan *et al.*, 2012), three-dimensional microfabrication, high-density optical data storage, optical limiting, stabilization and reshaping (He *et al.*, 2008). However, the basic structure-function relationships regarding enhanced TPA properties remain unclear. To further understand this important issue, we synthesized the two-photon absorption material with triphenylamine and pyridine units. This compound when dissolved in tetrahydrofuran shows absorption peak 430 nm. It must be noted that there is no linear absorption for the title chromophores beyond 800 nm. The laser pumped wavelength is 850 nm which is matching with absorption peak 430 nm, the linear absorption can be excluded because two-photon excitation via virtual intermediate state can proceed, consistent with the fact that the nonlinear absorption occurs at a frequency in the region of one-photon transparency. The title chromophore shows two-photon absorption fluorescence spectra at 546 nm when excited with 850 nm femtosecond laser pulses.

### Experimental

To a 100 mL methanol solution of 4-(diphenylamino) benzaldehyde (1.37 g, 5.0 mmol) was added 2-acetylpridine (0.61 g, 5.0 mmol) and 2% aqueous NaOH (0.44 g, 22 mL). The mixture was stirred for 2 h at room temperature (Scheme 1). The yellow precipitate formed was collected by filtration, and washed sequentially with water and methanol for three times, respectively.

### Refinement

All H atoms were placed in calculated positions with C—H equal 0.93 Å. They were included in the refinement in the riding model approximation with isotropic displacement parameters set equal to 1.2*U*_eq_(C).

### Crystal data

**Table:**

C_26_H_20_N_2_O	*F*(000) = 792
*M**_r_* = 376.44	*D*_x_ = 1.265 Mg m^−^^3^
Monoclinic, *P*2_1_/*c*	Melting point: 467 K
Hall symbol: -P 2ybc	Mo *K*α radiation, λ = 0.71073 Å
*a* = 12.0342 (3) Å	Cell parameters from 2266 reflections
*b* = 17.4069 (4) Å	θ = 2.5–21.1°
*c* = 9.4392 (2) Å	µ = 0.08 mm^−^^1^
β = 91.757 (2)°	*T* = 296 K
*V* = 1976.38 (8) Å^3^	Block, orange
*Z* = 4	0.20 × 0.20 × 0.18 mm

### Data collection

**Table:**

Bruker SMART 1000 CCD area-detector diffractometer	2210 reflections with *I* > 2σ(*I*)
Radiation source: fine-focus sealed tube	*R*_int_ = 0.046
graphite	θ_max_ = 25.0°, θ_min_ = 1.7°
φ and ω scans	*h* = −14→14
15074 measured reflections	*k* = −17→20
3488 independent reflections	*l* = −11→11

### Refinement

**Table:**

Refinement on *F*^2^	Secondary atom site location: difference Fourier map
Least-squares matrix: full	Hydrogen site location: inferred from neighbouring sites
*R*[*F*^2^ > 2σ(*F*^2^)] = 0.055	H-atom parameters constrained
*wR*(*F*^2^) = 0.167	*w* = 1/[σ^2^(*F*_o_^2^) + (0.0774*P*)^2^ + 0.1969*P*] where *P* = (*F*_o_^2^ + 2*F*_c_^2^)/3
*S* = 1.03	(Δ/σ)_max_ < 0.001
3488 reflections	Δρ_max_ = 0.17 e Å^−^^3^
263 parameters	Δρ_min_ = −0.16 e Å^−^^3^
0 restraints	Extinction correction: *SHELXTL* (Sheldrick, 2008), Fc^*^=kFc[1+0.001xFc^2^λ^3^/sin(2θ)]^-1/4^
Primary atom site location: structure-invariant direct methods	Extinction coefficient: 0.0084 (18)

### Special details

**Table:**

Experimental. ^1^H NMR (CDCl_3_; 400 MHz; TMS): ppm: 7.00-7.03 (m, 2H, Ar-H), 7.09-7.18 (m, 6H, Ar-H), 7.26-7.36 (m, 7H, Ar-H, pyr-H), 7.46-7.49 (m, 1H, Ar-H), 7.57, 7.59 (d, 1H, -CH=), 7.67, 7.69 (d, 1H, -CH=), 7.87-7.92 (m, 1H, pyr-H), 8.13-8.20 (m, 1H, pyr-H), 8.72, 8.74 (d, 1H, pyr-H)
Geometry. All esds (except the esd in the dihedral angle between two l.s. planes) are estimated using the full covariance matrix. The cell esds are taken into account individually in the estimation of esds in distances, angles and torsion angles; correlations between esds in cell parameters are only used when they are defined by crystal symmetry. An approximate (isotropic) treatment of cell esds is used for estimating esds involving l.s. planes.
Refinement. Refinement of F^2^ against ALL reflections. The weighted R-factor wR and goodness of fit S are based on F^2^, conventional R-factors R are based on F, with F set to zero for negative F^2^. The threshold expression of F^2^ > 2sigma(F^2^) is used only for calculating R-factors(gt) etc. and is not relevant to the choice of reflections for refinement. R-factors based on F^2^ are statistically about twice as large as those based on F, and R- factors based on ALL data will be even larger.

### Fractional atomic coordinates and isotropic or equivalent isotropic displacement parameters (Å^2^)

**Table:**

	*x*	*y*	*z*	*U*_iso_*/*U*_eq_	
C1	0.37944 (18)	0.15352 (15)	0.1848 (2)	0.0604 (6)	
C2	0.4306 (2)	0.22083 (15)	0.1472 (3)	0.0724 (7)	
H2	0.4013	0.2498	0.0721	0.087*	
C3	0.5246 (2)	0.24556 (18)	0.2199 (3)	0.0821 (8)	
H3	0.5590	0.2911	0.1939	0.099*	
C4	0.5675 (2)	0.2031 (2)	0.3304 (3)	0.0829 (9)	
H4	0.6305	0.2204	0.3803	0.099*	
C5	0.5181 (2)	0.13502 (18)	0.3686 (3)	0.0794 (8)	
H5	0.5484	0.1059	0.4428	0.095*	
C6	0.4231 (2)	0.11022 (15)	0.2955 (3)	0.0686 (7)	
H6	0.3890	0.0646	0.3210	0.082*	
C7	0.28491 (19)	0.11150 (14)	−0.0356 (2)	0.0592 (6)	
C8	0.1950 (2)	0.12480 (17)	−0.1262 (3)	0.0757 (8)	
H8	0.1298	0.1450	−0.0913	0.091*	
C9	0.2017 (3)	0.1082 (2)	−0.2684 (3)	0.0987 (11)	
H9	0.1402	0.1166	−0.3285	0.118*	
C10	0.2972 (3)	0.0796 (2)	−0.3223 (3)	0.1014 (11)	
H10	0.3008	0.0683	−0.4183	0.122*	
C11	0.3868 (3)	0.06786 (17)	−0.2342 (3)	0.0889 (9)	
H11	0.4526	0.0493	−0.2706	0.107*	
C12	0.3814 (2)	0.08311 (15)	−0.0914 (3)	0.0728 (7)	
H12	0.4432	0.0742	−0.0321	0.087*	
C13	0.18324 (18)	0.11941 (14)	0.1885 (2)	0.0615 (6)	
C14	0.1670 (2)	0.16653 (16)	0.3048 (3)	0.0728 (7)	
H14	0.2185	0.2047	0.3277	0.087*	
C15	0.0747 (2)	0.15704 (16)	0.3865 (3)	0.0735 (7)	
H15	0.0651	0.1894	0.4636	0.088*	
C16	0.10415 (18)	0.06364 (14)	0.1553 (2)	0.0630 (6)	
H16	0.1133	0.0319	0.0773	0.076*	
C17	0.01208 (19)	0.05508 (15)	0.2374 (3)	0.0662 (7)	
H17	−0.0406	0.0180	0.2125	0.079*	
C18	−0.00423 (19)	0.10043 (15)	0.3566 (3)	0.0634 (6)	
C19	−0.0996 (2)	0.08741 (15)	0.4450 (3)	0.0703 (7)	
H19	−0.1560	0.0565	0.4068	0.084*	
C20	−0.1147 (2)	0.11495 (15)	0.5740 (3)	0.0700 (7)	
H20	−0.0612	0.1476	0.6138	0.084*	
C21	−0.2123 (2)	0.09591 (15)	0.6561 (3)	0.0652 (7)	
C22	−0.21265 (18)	0.11975 (13)	0.8081 (3)	0.0593 (6)	
C23	−0.2974 (2)	0.09692 (16)	0.8930 (3)	0.0709 (7)	
H23	−0.3557	0.0674	0.8556	0.085*	
C24	−0.2956 (2)	0.11791 (17)	1.0331 (3)	0.0776 (8)	
H24	−0.3519	0.1023	1.0920	0.093*	
C25	−0.2104 (2)	0.16171 (17)	1.0843 (3)	0.0808 (8)	
H25	−0.2081	0.1782	1.1780	0.097*	
C26	−0.1279 (2)	0.18110 (19)	0.9946 (4)	0.0951 (10)	
H26	−0.0683	0.2096	1.0312	0.114*	
N1	0.28066 (15)	0.12811 (12)	0.1108 (2)	0.0678 (6)	
N2	−0.12745 (18)	0.16178 (14)	0.8578 (3)	0.0838 (7)	
O1	−0.29203 (14)	0.06052 (12)	0.60544 (19)	0.0871 (6)	

### Atomic displacement parameters (Å^2^)

**Table:**

	*U*^11^	*U*^22^	*U*^33^	*U*^12^	*U*^13^	*U*^23^
C1	0.0511 (12)	0.0775 (17)	0.0531 (13)	−0.0055 (12)	0.0112 (11)	−0.0105 (12)
C2	0.0659 (15)	0.0799 (19)	0.0721 (16)	−0.0092 (14)	0.0117 (13)	0.0002 (14)
C3	0.0770 (18)	0.088 (2)	0.082 (2)	−0.0209 (16)	0.0153 (16)	−0.0128 (16)
C4	0.0626 (16)	0.113 (2)	0.0733 (18)	−0.0179 (17)	0.0095 (14)	−0.0329 (18)
C5	0.0730 (17)	0.105 (2)	0.0602 (16)	0.0035 (17)	0.0014 (14)	−0.0096 (15)
C6	0.0686 (15)	0.0775 (17)	0.0601 (15)	−0.0093 (13)	0.0092 (13)	−0.0035 (13)
C7	0.0565 (13)	0.0688 (16)	0.0528 (13)	−0.0087 (11)	0.0108 (11)	−0.0030 (11)
C8	0.0592 (15)	0.102 (2)	0.0659 (17)	−0.0043 (14)	0.0051 (13)	0.0095 (15)
C9	0.083 (2)	0.149 (3)	0.0627 (18)	−0.030 (2)	−0.0072 (16)	0.0169 (18)
C10	0.104 (2)	0.141 (3)	0.0603 (17)	−0.049 (2)	0.0226 (18)	−0.0211 (18)
C11	0.085 (2)	0.102 (2)	0.082 (2)	−0.0166 (17)	0.0294 (17)	−0.0265 (17)
C12	0.0631 (15)	0.090 (2)	0.0660 (16)	0.0002 (13)	0.0146 (12)	−0.0121 (14)
C13	0.0496 (13)	0.0759 (17)	0.0596 (14)	−0.0029 (12)	0.0119 (11)	0.0001 (12)
C14	0.0644 (15)	0.0803 (19)	0.0751 (17)	−0.0125 (13)	0.0238 (13)	−0.0141 (14)
C15	0.0672 (16)	0.0824 (18)	0.0719 (16)	−0.0018 (14)	0.0205 (13)	−0.0113 (14)
C16	0.0568 (14)	0.0767 (17)	0.0559 (14)	−0.0016 (12)	0.0077 (11)	−0.0004 (12)
C17	0.0561 (13)	0.0777 (17)	0.0651 (15)	−0.0067 (12)	0.0088 (12)	0.0049 (13)
C18	0.0532 (13)	0.0729 (16)	0.0649 (15)	0.0024 (12)	0.0119 (11)	0.0076 (13)
C19	0.0601 (14)	0.0805 (18)	0.0710 (17)	0.0008 (13)	0.0165 (13)	0.0107 (13)
C20	0.0548 (14)	0.0858 (18)	0.0705 (16)	−0.0019 (12)	0.0173 (12)	0.0074 (14)
C21	0.0528 (14)	0.0754 (17)	0.0680 (16)	0.0014 (12)	0.0106 (12)	0.0091 (13)
C22	0.0477 (12)	0.0608 (15)	0.0699 (15)	0.0034 (11)	0.0119 (11)	0.0023 (12)
C23	0.0572 (14)	0.0858 (19)	0.0705 (16)	−0.0067 (13)	0.0139 (12)	−0.0055 (14)
C24	0.0697 (17)	0.090 (2)	0.0737 (18)	0.0008 (15)	0.0190 (14)	−0.0040 (15)
C25	0.0719 (17)	0.097 (2)	0.0740 (18)	0.0114 (16)	0.0064 (14)	−0.0146 (16)
C26	0.0718 (18)	0.113 (2)	0.101 (2)	−0.0186 (17)	0.0135 (17)	−0.0349 (19)
N1	0.0469 (11)	0.1011 (16)	0.0559 (12)	−0.0106 (10)	0.0117 (9)	−0.0101 (11)
N2	0.0690 (14)	0.0918 (17)	0.0919 (17)	−0.0185 (12)	0.0224 (12)	−0.0184 (14)
O1	0.0638 (11)	0.1250 (16)	0.0731 (12)	−0.0188 (11)	0.0105 (9)	−0.0031 (11)

### Geometric parameters (Å, °)

**Table:**

C1—C2	1.375 (3)	C13—N1	1.410 (3)
C1—C6	1.379 (3)	C14—C15	1.381 (3)
C1—N1	1.430 (3)	C14—H14	0.9300
C2—C3	1.374 (4)	C15—C18	1.392 (3)
C2—H2	0.9300	C15—H15	0.9300
C3—C4	1.366 (4)	C16—C17	1.380 (3)
C3—H3	0.9300	C16—H16	0.9300
C4—C5	1.379 (4)	C17—C18	1.394 (3)
C4—H4	0.9300	C17—H17	0.9300
C5—C6	1.386 (3)	C18—C19	1.457 (3)
C5—H5	0.9300	C19—C20	1.326 (3)
C6—H6	0.9300	C19—H19	0.9300
C7—C8	1.378 (3)	C20—C21	1.466 (3)
C7—C12	1.382 (3)	C20—H20	0.9300
C7—N1	1.414 (3)	C21—O1	1.225 (3)
C8—C9	1.377 (4)	C21—C22	1.493 (3)
C8—H8	0.9300	C22—N2	1.333 (3)
C9—C10	1.365 (4)	C22—C23	1.375 (3)
C9—H9	0.9300	C23—C24	1.372 (4)
C10—C11	1.357 (4)	C23—H23	0.9300
C10—H10	0.9300	C24—C25	1.355 (4)
C11—C12	1.377 (4)	C24—H24	0.9300
C11—H11	0.9300	C25—C26	1.366 (4)
C12—H12	0.9300	C25—H25	0.9300
C13—C14	1.389 (3)	C26—N2	1.335 (3)
C13—C16	1.389 (3)	C26—H26	0.9300
			
C2—C1—C6	119.9 (2)	C13—C14—H14	119.8
C2—C1—N1	120.6 (2)	C14—C15—C18	121.8 (2)
C6—C1—N1	119.5 (2)	C14—C15—H15	119.1
C3—C2—C1	120.4 (3)	C18—C15—H15	119.1
C3—C2—H2	119.8	C17—C16—C13	120.4 (2)
C1—C2—H2	119.8	C17—C16—H16	119.8
C4—C3—C2	119.8 (3)	C13—C16—H16	119.8
C4—C3—H3	120.1	C16—C17—C18	121.9 (2)
C2—C3—H3	120.1	C16—C17—H17	119.1
C3—C4—C5	120.6 (3)	C18—C17—H17	119.1
C3—C4—H4	119.7	C15—C18—C17	116.9 (2)
C5—C4—H4	119.7	C15—C18—C19	122.6 (2)
C4—C5—C6	119.5 (3)	C17—C18—C19	120.5 (2)
C4—C5—H5	120.2	C20—C19—C18	127.0 (3)
C6—C5—H5	120.2	C20—C19—H19	116.5
C1—C6—C5	119.8 (3)	C18—C19—H19	116.5
C1—C6—H6	120.1	C19—C20—C21	122.6 (3)
C5—C6—H6	120.1	C19—C20—H20	118.7
C8—C7—C12	118.5 (2)	C21—C20—H20	118.7
C8—C7—N1	121.4 (2)	O1—C21—C20	122.5 (2)
C12—C7—N1	120.1 (2)	O1—C21—C22	119.3 (2)
C9—C8—C7	120.1 (3)	C20—C21—C22	118.1 (2)
C9—C8—H8	119.9	N2—C22—C23	122.0 (2)
C7—C8—H8	119.9	N2—C22—C21	117.8 (2)
C10—C9—C8	120.9 (3)	C23—C22—C21	120.2 (2)
C10—C9—H9	119.5	C24—C23—C22	119.6 (3)
C8—C9—H9	119.5	C24—C23—H23	120.2
C11—C10—C9	119.3 (3)	C22—C23—H23	120.2
C11—C10—H10	120.4	C25—C24—C23	118.9 (3)
C9—C10—H10	120.4	C25—C24—H24	120.6
C10—C11—C12	120.7 (3)	C23—C24—H24	120.6
C10—C11—H11	119.7	C24—C25—C26	118.3 (3)
C12—C11—H11	119.7	C24—C25—H25	120.8
C11—C12—C7	120.5 (3)	C26—C25—H25	120.8
C11—C12—H12	119.8	N2—C26—C25	124.2 (3)
C7—C12—H12	119.8	N2—C26—H26	117.9
C14—C13—C16	118.6 (2)	C25—C26—H26	117.9
C14—C13—N1	119.2 (2)	C13—N1—C7	122.81 (19)
C16—C13—N1	122.2 (2)	C13—N1—C1	118.08 (18)
C15—C14—C13	120.4 (2)	C7—N1—C1	119.09 (18)
C15—C14—H14	119.8	C22—N2—C26	116.9 (2)
			
C6—C1—C2—C3	−0.5 (4)	C18—C19—C20—C21	−177.7 (2)
N1—C1—C2—C3	179.1 (2)	C19—C20—C21—O1	−8.4 (4)
C1—C2—C3—C4	−0.1 (4)	C19—C20—C21—C22	170.5 (2)
C2—C3—C4—C5	0.9 (4)	O1—C21—C22—N2	−176.4 (2)
C3—C4—C5—C6	−1.1 (4)	C20—C21—C22—N2	4.6 (3)
C2—C1—C6—C5	0.3 (4)	O1—C21—C22—C23	5.1 (4)
N1—C1—C6—C5	−179.3 (2)	C20—C21—C22—C23	−173.8 (2)
C4—C5—C6—C1	0.5 (4)	N2—C22—C23—C24	0.3 (4)
C12—C7—C8—C9	1.5 (4)	C21—C22—C23—C24	178.7 (2)
N1—C7—C8—C9	179.8 (3)	C22—C23—C24—C25	0.8 (4)
C7—C8—C9—C10	−1.0 (5)	C23—C24—C25—C26	−2.0 (4)
C8—C9—C10—C11	−0.4 (5)	C24—C25—C26—N2	2.3 (5)
C9—C10—C11—C12	1.2 (5)	C14—C13—N1—C7	−150.5 (2)
C10—C11—C12—C7	−0.7 (4)	C16—C13—N1—C7	31.4 (4)
C8—C7—C12—C11	−0.6 (4)	C14—C13—N1—C1	30.6 (3)
N1—C7—C12—C11	−179.0 (2)	C16—C13—N1—C1	−147.4 (2)
C16—C13—C14—C15	1.0 (4)	C8—C7—N1—C13	34.6 (4)
N1—C13—C14—C15	−177.2 (2)	C12—C7—N1—C13	−147.1 (2)
C13—C14—C15—C18	0.4 (4)	C8—C7—N1—C1	−146.6 (2)
C14—C13—C16—C17	−0.6 (4)	C12—C7—N1—C1	31.7 (3)
N1—C13—C16—C17	177.5 (2)	C2—C1—N1—C13	−119.3 (3)
C13—C16—C17—C18	−1.1 (4)	C6—C1—N1—C13	60.2 (3)
C14—C15—C18—C17	−2.0 (4)	C2—C1—N1—C7	61.8 (3)
C14—C15—C18—C19	177.4 (2)	C6—C1—N1—C7	−118.6 (2)
C16—C17—C18—C15	2.4 (4)	C23—C22—N2—C26	−0.1 (4)
C16—C17—C18—C19	−177.0 (2)	C21—C22—N2—C26	−178.5 (2)
C15—C18—C19—C20	−13.4 (4)	C25—C26—N2—C22	−1.2 (5)
C17—C18—C19—C20	165.9 (3)		

### Hydrogen-bond geometry (Å, °)

**Table:**

*D*—H···*A*	*D*—H	H···*A*	*D*···*A*	*D*—H···*A*
C5—H5···O1^i^	0.93	2.55	3.404 (3)	153
C6—H6···O1^ii^	0.93	2.58	3.505 (3)	179

Symmetry codes: (i) *x*+1, *y*, *z*; (ii) −*x*, −*y*, −*z*+1.
